# Histone ZmH2B regulates resistance to the Southern corn leaf blight pathogen *Bipolaris maydis* in maize

**DOI:** 10.1186/s12870-025-07020-9

**Published:** 2025-08-19

**Authors:** Ying Ding, Hongliang Wu, Na Ning, Haiyue Yu, Chaotian Liu, Ruoya Lv, Shu Li, Junjie Hao, Xintao Wang, Xuecai Zhang, Xin Xie, Xinfeng Li, Wende Liu, Zhiqiang Li

**Affiliations:** 1 College of Plant Protection, Shanxi Agricultural University, Taigu, 030801 China; 2 State Key Laboratory for Biology of Plant Diseases and Insect Pests, Institute of Plant Protection, Chinese Academy of Agricultural Sciences, Beijing, 100193 China; 3 Institute of Plant Protection, Henan Academy of Agricultural Sciences, Zhengzhou, 450002 China; 4 International Maize and Wheat Improvement Center (CIMMYT), 56237 Texcoco, Mexico; 5 Institute of Crop Sciences, Chinese Academy of Agricultural Sciences (CAAS)CIMMYT-China Office, 12 Zhongguancun South Street, Beijing, 10008L China; 6 Nanfan Research InstituteCAAS, Sanya, 572024 China; 7 College of Agriculture, Guizhou University, Guiyang, 550025 China

**Keywords:** Maize, *ZmH2B*, *Bipolaris maydis*, Virus-induced gene silencing and overexpression, Transcriptome analysis

## Abstract

**Background:**

H2B histones play crucial roles in plant responses to biotic stress. However, to date, most research on H2B histones has focused on their roles in post-translational modification, and studies specifically investigating the intrinsic properties of these histones remain relatively limited. Here we identified the ZmH2B in maize (*Zea mays*) and investigated its role in the response of maize to infection by the Southern corn leaf blight pathogen *Bipolaris maydis*.

**Result:**

In this study, a nucleus-localized ZmH2B was identified from maize. To characterize the role of this histone in disease resistance, we employed virus-induced gene silencing (VIGS) and transient overexpression (VOX) to generate *ZmH2B*-silenced (FoMV:*ZmH2B*) and *ZmH2B*-overexpressing (FoMV:*ZmH2B*-VOX) lines. FoMV:*ZmH2B* lines showed enhanced *B. maydis* infection and an inhibited chitin-induced reactive oxygen species burst, whereas FoMV:*ZmH2B*-VOX lines exhibited the opposite effects. Furthermore, *ZmH2B* overexpression induced the expression of various pathogenesis-related genes, suggesting that these genes enhance resistance against *B. maydis*. Transcriptome analysis of *ZmH2B*-silenced plants revealed that the differentially expressed genes were predominantly enriched in photosynthesis-related pathways, pointing to a role for photosynthesis in *B. maydis* resistance.

**Conclusions:**

These results suggest that ZmH2B positively regulates maize resistance to *B. maydis*.

**Supplementary Information:**

The online version contains supplementary material available at 10.1186/s12870-025-07020-9.

## Background

In eukaryotic cells, the regulation of chromatin structure within the genome is primarily governed by the assembly of nucleosome complexes comprising histones and DNA [1]. Five major histone families play essential and distinct roles in this process: H1, H2A, H2B, H3, and H4. These histone families serve as fundamental components contributing to the organization and regulation of DNA within the nucleus [2]. Among the five families, H2A, H2B, H3, and H4 function as core histones, forming the central structure around which DNA winds to create the nucleosome core particle. By contrast, H1 acts as linker histone, binding to the DNA segments between nucleosomes to help organize chromatin into higher-order structures. Notably, while H4 has a relatively stable and conserved form across different organisms, H1, H2A, H2B, and H3 have multiple variants [3]. These variants often have distinct biochemical properties and play unique roles in various cellular processes related to DNA metabolism and gene regulation. In vitro, diverse histone variants endow nucleosomes with a wide range of stability levels, representing a fundamental regulatory mechanism governing chromatin structure and function [4]. These four families of core histones comprise two structurally and functionally distinct structural domains: histone tails and folds [5]. The 20–35 N-terminal residues (~ 20% of the total amino acids) of each histone form the histone tail, and approximately 80–90 residues in each histone comprise the histone fold, which mediates interactions with other histones [6] and DNA [7, 8]. Histone tails and folds are subject to extensive post-translational modifications, which are involved in transcriptional activation, silencing, chromatin assembly, and DNA replication [9, 10].

The fundamental subunit of chromatin is the nucleosome, which consists of an octameric core of histones H2A, H2B, H3 and H4, around which approximately 145–147 base pairs (bp) of DNA are wound [11, 12]. Nucleosomes are critical to various chromatin-related processes, including transcription, DNA replication, repair, and recombination [13, 14]. The repetitive nucleosome core particles subsequently assemble into higher-order chromatin structures. The stability of these structures is maintained by the linker histone H1 and the intervening linear DNA. Nucleosomes facilitate the formation of higher-order chromatin structures through DNA bending or the creation of higher-order helices. As the primary packaging units of DNA within the nucleus, nucleosomes are crucial factors that determine DNA accessibility [15].

Histone H2B proteins play crucial roles in plants exposed to biotic stress by altering the levels of histone modifications on the chromatin of defense response-related genes. These modifications, including acetylation, methylation, and mono-ubiquitination, are likely involved in plant defense responses [16, 17]. Histone modifications modulate the expression of defense genes and are key components of the signaling and defense processes that occur during the interactions between plants and pathogenic fungi. For example, histone H2B monoubiquitination (H2Bub1), catalyzed by HUB1, confers resistance against the necrotrophic fungi *Botrytis cinerea* and *Alternaria brassicicola* in *Arabidopsis* (*Arabidopsis thaliana*) [18]. HUB1 acts independently of jasmonic acid (JA) but integrates the ethylene and salicylic acid (SA) pathways [19]. Mechanistically, HUB1 interacts with MED21 to bridge H2Bub1-modified chromatin to RNA polymerase II, enhancing the transcription of defense genes (*PDF1.2*, *WRKY33*, *EXTENSIN*) [20–22]. ChIP analysis revealed the pathogen-induced co-enrichment of H2Bub1 and MED21 at these promoters. This H2Bub1-MED21 axis orchestrates chromatin remodeling and defense networks that are critical for resistance.

H2Bub1 is catalyzed by the RING E3 ligases histone mono-ubqutination1 (HUB1) and HUB2 [23, 24]. To date, numerous reports have been published regarding HUB1 and HUB2. In tomato (*Solanum lycopersicum*), SIHUB1 and SIHUB2 positively regulate disease resistance to *B. cinerea* by balancing the SA and the JA- and ethylene-mediated signaling pathways [25]. In rice (*Oryza sativa*), OsHUB1 and OsHUB2 form a complex to regulate PL11-INTERACTING PROTEIN 6 (SPIN6)/OsRac1-mediated defense responses against *Magnaporthe oryza*e [26]. In *Arabidopsis*, H2Bub1 is essential for the defense response to *Verticillium dahliae* toxin. This modification regulates the NADPH oxidase RbohD, which affects H_2_O_2_ production, and modulates the expression of components in the PTP-MPK3/6-WRKY pathway [27, 28]. The *Arabidopsis hub1* mutant shows differential regulation of genes involved in meristem development [29], plant metabolism [29], photosynthesis [30], and exhibits an auxin-deficient phenotype [31].

Most studies of histone H2B performed to date have focused on its modification-related functions. In-depth investigations have revealed how histone H2B modifications affect various cellular processes. However, a notable gap exists in our knowledge of changes in these modifications following infection by pathogenic fungi. Therefore, in this study, we performed transcriptome sequencing of the maize (*Zea mays*) inbred line B73 following spray inoculation with the Southern corn leaf blight pathogen *Bipolaris maydis. ZmH2B* exhibited a marked response to *B. maydis* infection. Our findings from *ZmH2B*-silenced and *ZmH2B-*overexpressing lines highlight the potential importance of *ZmH2B* in plant–pathogen interactions and address a critical knowledge gap in histone H2B research, particularly regarding its epigenetic regulatory functions during host–pathogen interactions. Further study of *ZmH2B* could provide a more comprehensive understanding of how histone H2B functions during pathogenic fungi infection, offering insight into plant defense mechanisms and laying the foundation for developing new agricultural disease control strategies.

## Results

### Bioinformatics analysis of *ZmH2B*

**Fig. 1:**
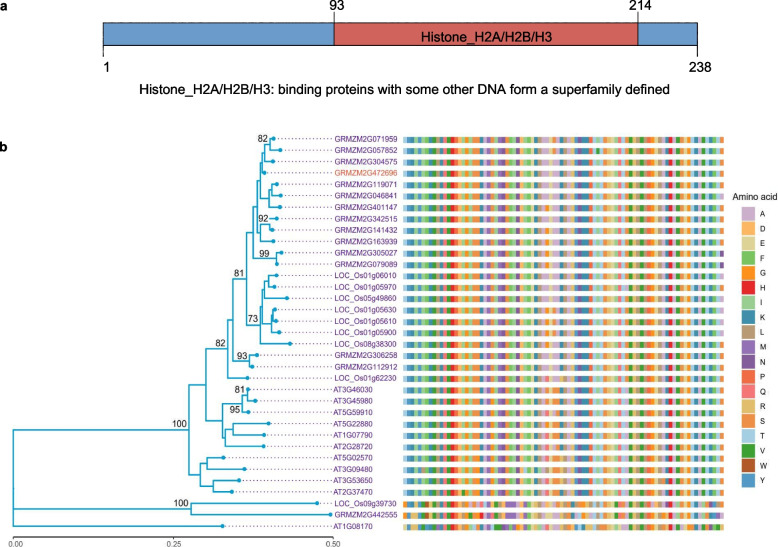
The bioinformatics analysis of *ZmH2B.***a** Protein structure diagram of ZmH2B. ZmH2B protein structure was obtained in UniProt and protein structure mapping was performed using IBS 2.0. **b** Phylogenetic tree analysis and multiple sequence comparison of ZmH2B. A total of 34 homologous gene protein sequences from maize, *Arabidopsis thaliana*, and rice were used to construct a phylogenetic tree using the neighbor-joining method in MEGA 7, and multiple sequence comparisons were performed using ggmsa [32]. ZmH2B (GRMZM2G472696) is highlighted in red

*ZmH2B* (GRMZM2G472696) is a 717 bp gene encoding a protein of 238 amino acids belonging to the histone H2B family (Supplementary Fig. 1). ZmH2B harbors a Histone_H2A/H2B/H3 structural domain, which is central to several fundamental cellular processes, predominantly transcriptional regulation, DNA repair, DNA replication, and the maintenance of chromosome stability (Fig. 1a). To explore the evolutionary relationships and identify potential homologs of *ZmH2B,* we compared the amino acid sequence of ZmH2B to other sequences in the NCBI (National Center for Biotechnology Information) database via BLAST analysis. This analysis yielded a total of 14 homologous genes in maize. Guided by Jiang’s research [1], we selected 9 H2B sequences from rice and 11 H2B sequences from *Arabidopsis* and used these to construct a phylogenetic tree and conduct multiple sequence alignment. The differences between H2B sequences were relatively minor (Fig. 1b). This conservation suggests that the functions of H2B proteins are evolutionarily conserved, which could have significant implications for understanding the biological processes related to H2B in maize and other plants.

### *ZmH2B *is upregulated in response to *B. maydis* infection

**Fig. 2:**
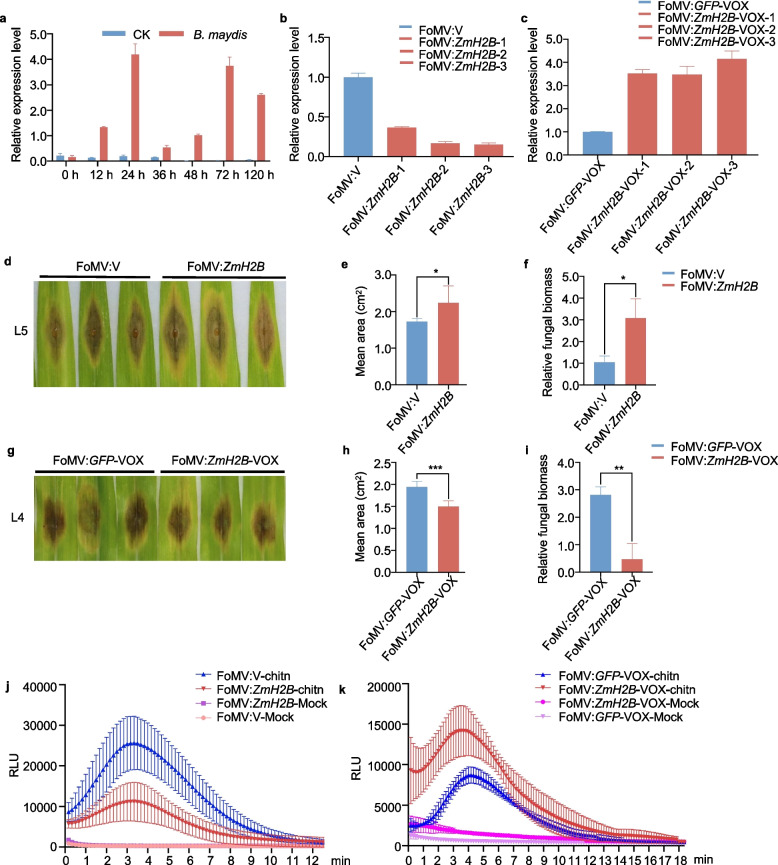
*ZmH2B* positively regulates *B. maydis* infection. **a** RT-qPCR results demonstrated that *ZmH2B* positively responded to *B. maydis* infection. Samples were taken at 0 h, 12 h, 24 h, 36 h, 48 h, 72 h, and 120 h after infection of B73 with *B. maydis*, and the control group was treated with water. RT-qPCR was performed with *ZmActin* as the internal reference gene. Values for each group are standard deviations of three biological replicates. **b** Silencing efficiency of FoMV:*ZmH2B* plants was determined after injection. **c** Transient overexpression efficiency of FoMV:*ZmH2B*-VOX plants was determined after injection. **d e f** Identification of disease-resistant phenotypes, lesion area, and fungal biomass of *ZmH2B*-silenced material inoculated in vitro with *B. maydis*. **g** **h** **i** Identification of disease resistance phenotypes, lesion area, and fungal biomass statistics of *ZmH2B*-overexpressed material in vitro inoculation *B. maydis*. **j** **k** ROS assay of FoMV:*ZmH2B*, FoMV:*ZmH2B*-VOX plants. Data contain mean ± standard error of three replicates (* *p* ≤ 0.05; ** *p* ≤ 0.01; *** *p* ≤ 0.001)

To gain a deeper understanding of the function of ZmH2B, we examined the expression dynamics of *ZmH2B* in maize plants inoculated with *B. maydis* across six stages of infection via RT-qPCR: 12, 24, 36, 48, 72, and 120 h post-inoculation (hpi). *ZmH2B* expression was significantly upregulated during the initial stage of infection, specifically at 24 hpi (Fig. 2a), indicating that *ZmH2B* responds positively to *B. maydis* infection.

Subsequently, to comprehensively explore the role of *ZmH2B* in resistance against *B. maydis*, we developed *ZmH2B*-silenced and *ZmH2B*-overexpressing maize plants through virus-induced gene silencing (VIGS) and overexpression (VOX), both mediated by foxtail mosaic virus (FoMV). When we injected the plants with *Agrobacterium* suspensions harboring the VIGS and VOX constructs and measured the relative expression level of *ZmH2B*, *ZmH2B* transcripts were barely detectable in *ZmH2B*-silenced plants and were significantly less abundant than in the FoMV:V control plants. Specifically, *ZmH2B* transcript levels in the three silenced maize lines, FoMV:*ZmH2B*−1, FoMV:*ZmH2B*−2, and FoMV:*ZmH2B*−3 plants, were 63%, 83%, and 85% lower, respectively, than those in FoMV:V (Fig. 2b). Conversely, in *ZmH2B*-overexpressing plants, *ZmH2B* was markedly upregulated, with *ZmH2B* transcript levels in the three overexpression lines, FoMV:*ZmH2B*-VOX-1, FoMV:*ZmH2B*−2, and FoMV:*ZmH2B*-VOX-3, being 3.5-, 3.5-, and 4.2-fold higher, respectively (Fig. 2c).

To obtain more *ZmH2B*-silenced and -overexpressing plants for subsequent phenotyping, we performed rub inoculation of maize leaves and measured the relative expression levels of *ZmH2B*. The transcript levels of this gene were significantly reduced in FoMV:*ZmH2B*−1, FoMV:*ZmH2B*−2, and FoMV:*ZmH2B*−3 compared to FoMV:V plants, with decreases of 45%, 46%, and 36%, respectively (Supplementary Fig. 2a). Conversely, in *ZmH2B*-overexpressing plants, *ZmH2B* was markedly upregulated. Compared to FoMV:*GFP*-VOX plants, the transcript levels of *ZmH2B* in FoMV:*ZmH2B*-VOX-1, FoMV:*ZmH2B*−2, and FoMV:*ZmH2B*-VOX-3 increased by 1,841-, 400-, and 1,829-fold, respectively (Supplementary Fig. 2b). Subsequently, we separately inoculated the *ZmH2B*-silenced lines and the *ZmH2B*-overexpressing lines with *B. maydis* to assess their disease-resistance phenotypes. The lesion area and relative fungal biomass of *B. maydis* were significantly higher in *ZmH2B*-silenced plants than in FoMV:V plants (Fig. 2d-f). Similarly, the lesion area and relative fungal biomass of *B. maydis* were significantly lower in *ZmH2B*-overexpressing plants than in FoMV:*GFP*-VOX plants (Fig. 2g-i). These results strongly suggest that ZmH2B positively regulates resistance against *B. maydis* in maize.

To further validate the role of ZmH2B in maize immunity, we examined chitin-induced reactive oxygen species (ROS) bursts in FoMV:V, FoMV:*ZmH2B*, FoMV:*GFP*-VOX, and FoMV:*ZmH2B*-VOX plants. The ROS burst was diminished in FoMV:*ZmH2B* plants compared to FoMV:V plants (Fig. 2j), whereas it was elevated in FoMV:*ZmH2B*-VOX plants compared to FoMV:*GFP*-VOX plants (Fig. 2k). Moreover, the ROS bursts in FoMV:*ZmH2B* and FoMV:*ZmH2B*-VOX plants reached their peaks at 3.5 min and 4 min after chitin treatment, respectively. These results further confirm the notion that ZmH2B positively regulates maize immunity, providing a more complete understanding of how ZmH2B contributes to plant defense against *B. maydis* infection.

### ZmH2B modulates the expression levels of pathogenesis-related genes

**Fig. 3:**
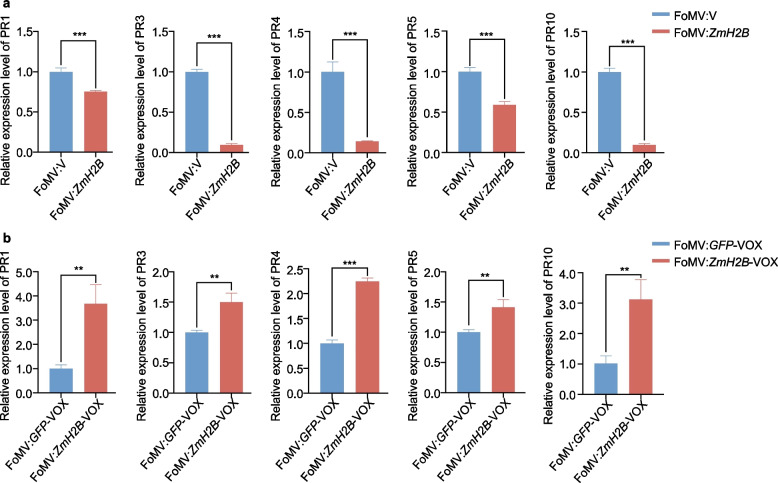
The expression of PR genes in *ZmH2B*-silenced materials FoMV:*ZmH2B* and *ZmH2B*-overexpressed materials FoMV:*ZmH2B*-VOX. **a** RT-qPCR analyses showing the expression of *ZmPR1*, *ZmPR3*, *ZmPR4*, *ZmPR5* and *ZmPR10* in FoMV:*ZmH2B*. **b** RT-qPCR analyses showing the expression of *ZmPR1*, *ZmPR3*, *ZmPR4*, *ZmPR5* and *ZmPR10* in FoMV:*ZmH2B*-VOX. Data contain mean ± standard error of three replicates (* *p* ≤ 0.05; ** *p* ≤ 0.01; *** *p* ≤ 0.001)

To further explore the role of ZmH2B in the defense mechanism against *B. maydis*, we focusing on the expression levels of maize pathogenesis-related (PR) genes. These genes included *ZmPR1*, *ZmPR3*, *ZmPR4*, *ZmPR5*, and *ZmPR10*, which are associated with plant defense responses [33, 34]. We conducted our analysis on both *ZmH2B*-silenced and *ZmH2B*-overexpressing maize plants. For the *ZmH2B*-silenced group, we compared the expression of these PR genes in *ZmH2B*-silenced maize plants with that in FoMV:V plants. *ZmPR1*, *ZmPR3*, *ZmPR4*, *ZmPR5*, and *ZmPR10* were downregulated in *ZmH2B*-silenced (FoMV:*ZmH2B*) plants, indicating that the absence of ZmH2B led to a decrease in the expression of these crucial PR genes (Fig. 3a). On the other hand, all five genes were significantly upregulated in FoMV:*ZmH2B*-VOX compared to FoMV:*GFP*-VOX plants (Fig. 3b). We then analyzed the expression levels of these PR genes following infection with *B. maydis.* All five were downregulated in FoMV:*ZmH2B* plants but significantly upregulated in FoMV:*ZmH2B*-VOX compared with FoMV:*GFP*-VOX plants (Supplementary Fig. 3). These results demonstrate that ZmH2B regulates the expression of PR genes. The positive correlation between *ZmH2B* transcript levels and the expression of these PR genes further validates the notion that *ZmH2B* positively responds to *B. maydis* infection. This response not only affects *ZmH2B*’s own expression but also has cascading effects on the expression of other PR genes involved in maize defense against *B. maydis*, providing a more in-depth understanding of the complex defense mechanism in maize.

### ZmH2B localizes to the nucleus

**Fig. 4:**
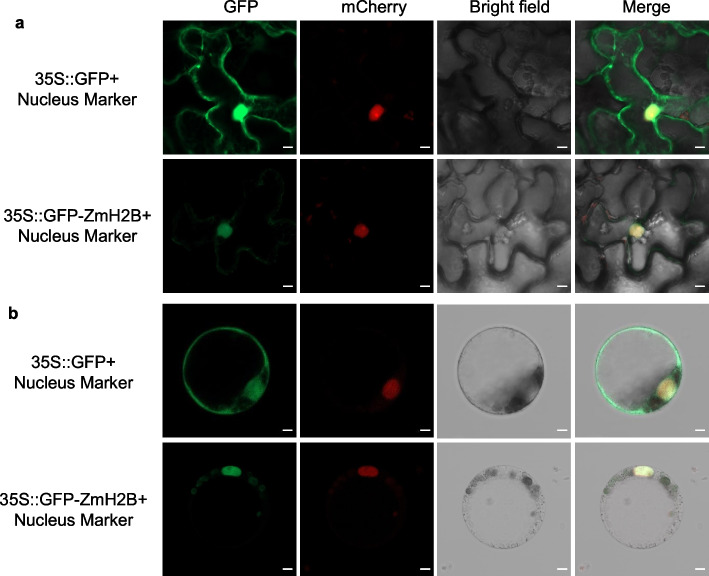
The ZmH2B protein is localized in the nucleus**. a** GFP, GFP-ZmH2B, and cell nucleus marker of mCherry fusion proteins transformed into *Agrobacterium tumefaciens* and infiltrated into *N. benthamiana* leaves. Confocal microscopy images were taken at 36 h after infiltration. Scale bars = 5 μm. **b** GFP, GFP-ZmH2B, and cell nucleus marker of mCherry fusion proteins transformed into maize protoplasts. Confocal microscopy images were observed after 12 h of incubation protected from light. Scale bars = 5 μm

Understanding the subcellular localization of a protein is crucial for deciphering its biological functions. To determine the sites of ZmH2B expression within the plant cell, we carried out transient expression assays using ZmH2B-green fluorescent protein (GFP) fusion protein in *Nicotiana benthamiana* leaves and maize protoplasts. After introducing the GFP-ZmH2B construct into these cells via transient transfection, we employed laser confocal microscopy (Zeiss Confocal LSM 980) to observe the subcellular localization of the fusion protein. As a control, we detected the GFP empty construct in both the cytoplasm and nucleus. In contrast, the GFP-ZmH2B fusion protein precisely co-localized with the red fluorescence emitted by a nucleus-specific marker in both* N. benthamiana* leaves and maize protoplasts (Fig. 4a-b), providing strong evidence that ZmH2B localizes to the nucleus.

### Transcriptomic analysis of *ZmH2B*-silenced plants

**Fig. 5:**
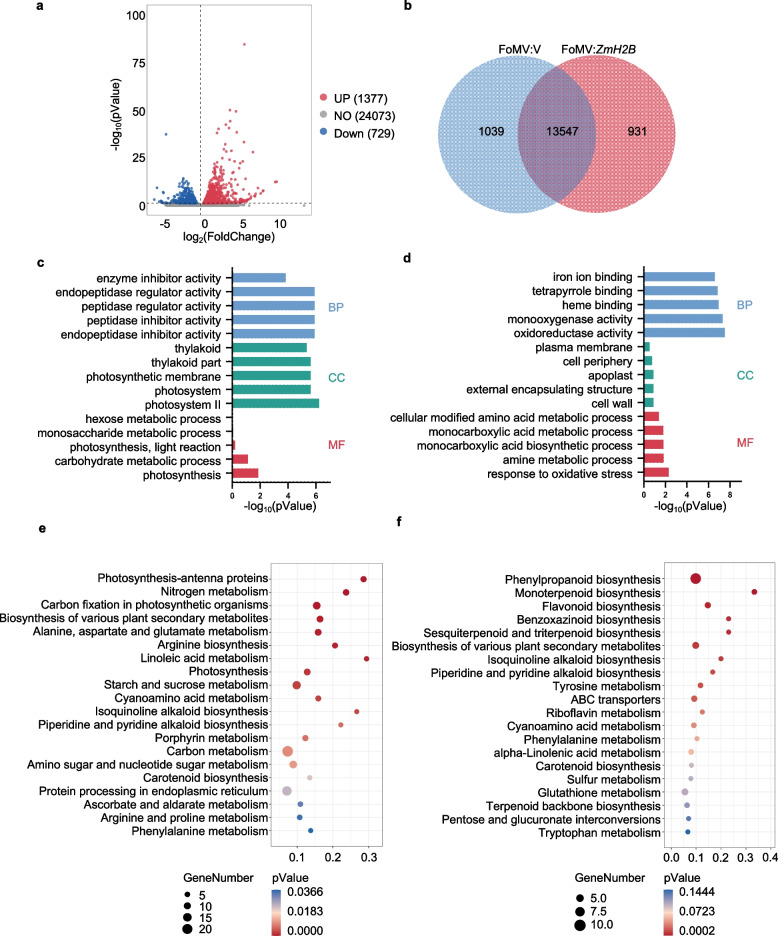
Analysis of transcriptome data from *ZmH2B*-silenced plants. **a** Volcano plot for the analysis of differentially expressed genes in *ZmH2B*-silenced plants (*p*-Value ≤ 0.05,|log2FoldChange|≥ 1). **b** Venn diagram of the differentially expressed genes in *ZmH2B*-silenced plants. **c** GO enrichment pathways of the up-regulated differentially expressed genes in *ZmH2B*-silenced plants. **d** GO enrichment pathways of the down-regulated differentially expressed genes in *ZmH2B*-silenced plants. **e** KEGG enrichment pathways of the up-regulated differentially expressed genes in *ZmH2B*-silenced plants. **f** KEGG enrichment pathways of the down-regulated differentially expressed genes in *ZmH2B*-silenced plants

Unraveling the regulatory pathway through which ZmH2B contributes to maize resistance against *B. maydis* is essential for understanding the plant–pathogen interaction mechanism. We therefore performed comprehensive transcriptome analysis using *ZmH2B*-silenced plants and control FoMV:V plants. All *ZmH2B*-silenced plants showed high silencing efficiency (Supplementary Fig. 4a). Transcriptome analysis revealed that most genes exhibited stable expression (Fig. 5a, gray dots), with 2,106 differentially expressed genes (DEGs) identified between FoMV:V and FoMV:*ZmH2B*. Of these, 1,377 genes were upregulated (red dots) and 729 were downregulated (blue dots), showing significant changes in transcription. A total of 14,586 genes were detected in control (FoMV:V) plants, 14,478 genes were detected in *ZmH2B*-silenced plants (FoMV:*ZmH2B*), and 13,547 genes were co-expressed under both conditions (Fig. 5b).

To gain deeper insights into the functions associated with these DEGs, we subjected the 1,944 DEGs in *ZmH2B-*silenced plants to Gene Ontology (GO) enrichment analysis. The genes were classified into three categories: Biological process (BP), Cellular component (CC), and Molecular function (MF). When comparing FoMV:*ZmH2B* with FoMV:V plants, the upregulated genes were predominantly enriched in pathways related to photosystem II (PSII), endopeptidase inhibitor activity, peptidase inhibitor activity, endopeptidase regulator activity, enzyme inhibitor activity, endopeptidase regulator activity, and enzyme inhibitor activity (Fig. 5c). These results suggest that *ZmH2B* silencing enhances processes related to photosynthesis-associated functions and enzyme-inhibition-related activities. Meanwhile, the downregulated genes were mainly enriched in pathways such as oxidoreductase activity, phosphoric ester hydrolase activity, monooxygenase activity, heme binding, tetrapyrrole binding, and iron ion binding (Fig. 5d). We also performed heatmap analysis of genes involved in the protein heterodimerization activity pathway enriched in *ZmH2B-*silenced plants, in which a total of 8 genes were upregulated and 3 genes were downregulated (Supplementary Fig. 4b). These findings suggest that silencing *ZmH2B* positively regulates photosynthesis while negatively regulating respiration in maize.

Subsequently, to identify the major causal pathways and key genes, we performed Kyoto Encyclopedia of Genes and Genomes (KEGG) enrichment analysis [35] of the 1,944 DEGs in FoMV:*ZmH2B*. Enrichment analysis of upregulated genes identified 83 significantly enriched pathways, from which the top 20 (based on a significance threshold of *p*-value < 0.05) were selected to construct a bubble plot. The photosynthesis-antenna protein pathway, with the lowest *p*-value, was the most significantly enriched. Among these pathways, the carbon metabolism pathway harbored the highest gene count (23 genes, the largest proportional enrichment), followed by the protein processing in endoplasmic reticulum (19 genes) and starch and sucrose metabolism (15 genes) pathways, which exhibited substantial gene enrichment (Fig. 5e). These results further support the notion that *ZmH2B* silencing affects photosynthesis-related pathways.

Enrichment analysis of downregulated genes identified 103 significantly enriched pathways, and the top 54 pathways were represented. We selected the top 20 pathways (based on a *p*-value < 0.05 threshold) to construct a bubble plot. The phenylpropanoid biosynthesis pathway, with the lowest *p*-value, exhibited the most significant enrichment and simultaneously harbored the highest number of enriched genes (12 genes). This was followed by the biosynthesis of various plant secondary metabolites and glutathione metabolism pathways, each containing 6 enriched genes (Fig. 5f). These pathways are involved in the production of secondary metabolites, suggesting that ZmH2B might play a role in regulating the biosynthesis of these important compounds, which could be associated with maize defense mechanisms against *B. maydis*. Overall, these transcriptome data provide valuable clues about the complex regulatory network involving ZmH2B during the response of maize to the pathogen.

### Validation of transcriptomic data from *ZmH2B*-silenced plants

**Fig. 6:**
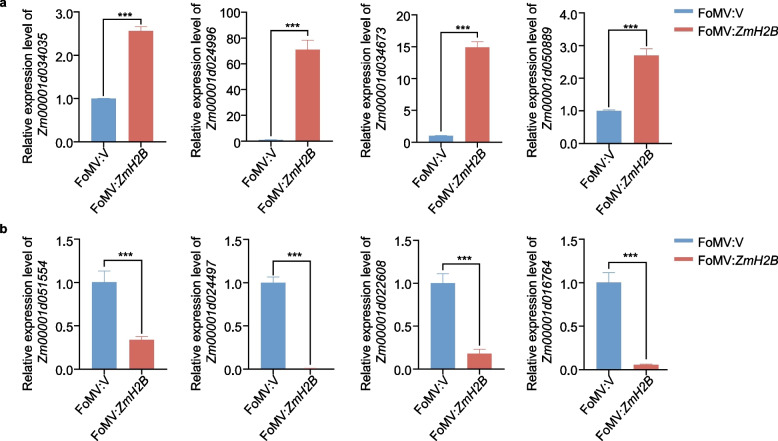
The RT-qPCR confirm the transcriptome analysis result in *ZmH2B*-silenced plants. RT-qPCR detection of the relative expression levels of the 8 randomly selected genes in FoMV:*ZmH2B* and FoMV:V. **a** Up-regulated expression gene validation. **b** Down-regulated expression gene validation. Data contain mean ± standard error of three replicates (* p ≤ 0.05; ** p ≤ 0.01; *** p ≤ 0.001)

To verify the integrity of the transcriptome data obtained from the *ZmH2B*-silenced plants (FoMV:*ZmH2B*) and the control plants (FoMV:V), we randomly selected both upregulated and downregulated genes from the list of DEGs identified by transcriptome analysis for RT-qPCR. Among the upregulated genes, Zm00001d034035, Zm00001d024996, Zm00001d034673, and Zm00001d050889 exhibited significantly increased expression in FoMV:*ZmH2B* compared to FoMV:V (Fig. 6a). Among the downregulated genes, Zm00001d051554, Zm00001d022608, Zm00001d024497, and Zm00001d016764 showed marked decreases in expression in FoMV:*ZmH2B* compared to FoMV:V (Fig. 6b). Importantly, the expression trends of these randomly selected genes precisely matched those indicated by the original transcriptome data, providing strong evidence for the reliability of the transcriptome data.

## Discussion

When conducting protein structure prediction for ZmH2B, we discovered that the Histone_H2A/H2B/H3 domain in ZmH2B is also present in certain transcription factors, such as OsNF-YC2 (OsHAP5C) in rice. Nuclear factor Y (NF-Y) transcription factors play important roles in regulating multiple biological processes in plants, including embryonic development, flowering time, chloroplast biogenesis, seed reserve accumulation, and stress tolerance [36], by forming heterotrimeric complexes with other proteins. Under long-day conditions, OsNF-YC2 and OsNF-YC4 repress flowering in rice by directly associating with OsNF-YB8, OsNF-YB10, and OsNF-YB11 to modulate the photoperiodic flowering response [37]. Specifically, OsNF-YC2 delays tasseling, possibly by downregulating *Ehd1*, *Hd3a*, and *RFT1* expression [38]. Furthermore, Ghd8 can interact with OsNF-YC2 and Ghd7 and form a complex in vivo and in vitro. This Ghd7–Ghd8–OsNF-YC2 ternary complex directly binds to the promoter of *Hd3a*, leading to the downregulation of *Ehd1*, *Hd3a* and *RFT1* expression, ultimately resulting in delayed tasseling [39].

In this study, we determined that *ZmH2B* responds significantly to *B. maydis* infection and positively regulates maize resistance to this pathogen. Specifically, PR genes were downregulated in *ZmH2B*-silenced plants but upregulated in *ZmH2B*-overexpressing plants. The positive correlation between *ZmH2B* transcript levels and the expression of these PR genes further confirms the finding that *ZmH2B* positively responds to *B. maydis* infection. This response not only affects the expression of *ZmH2B* itself but also has cascading effects on the expression of other key genes involved in maize defense against the pathogen, providing a more in-depth understanding of the complex defense mechanisms in maize. However, the regulatory mechanism by which ZmH2B confers disease resistance remains to be elucidated.

Analysis of the transcriptomic data revealed that many DEGs were enriched in the PSII-related pathway following *ZmH2B* silencing. We hypothesize that this enrichment is closely related to *ZmH2B*-mediated maize immunity. PSII is a multi-subunit chlorophyll-protein complex embedded within the thylakoid membranes of the chloroplast that plays a crucial role in photosynthesis [40, 41]. Chloroplasts have key roles in both plant growth and disease resistance [42] and are the sites where precursors of the plant hormones SA and JA are produced [43]. In fact, SA and JA biosynthesis and signaling are tightly linked to transcriptional reprogramming, as exemplified by the *Arabidopsis* histone methyltransferase SDG8. SDG8 regulates JA pathway genes, and the loss-of-function mutant *sdg8-1* shows compromised resistance to the necrotrophic fungal pathogens *Alternaria brassicicola* and *Botrytis cinerea* [44]. Certain chloroplast proteins also participate in plant immune responses. For example, the calcium-sensing receptor induces the specific accumulation of Ca^2+^ and is involved in pathogen-associated molecular patter (PAMP)-induced basal resistance and R-gene-mediated hypersensitivity, thereby enabling chloroplast-mediated transcriptional reprogramming during plant immune responses [45]. Qi et al. [46] determined that the expression of the chloroplast elongation factor *StTuA/B* enhanced both photosynthesis and yield in potato (*Solanum tuberosum*) and increased resistance to potato late blight. The effector Pi22926 inhibits the phosphorylation of StTuA/B by the mitogen-activated protein kinase StMAP3Kβ2, leading to its retention in the cytoplasm and subsequent degradation, thus suppressing the plant immune response. In summary, the enrichment of DEGs in the PSII pathway after *ZmH2B* silencing, combined with the multifaceted roles of chloroplasts in plant hormone production and protein-mediated immune responses, suggests that PSII- and chloroplast-related photosynthetic processes represent integral components of the *ZmH2B*-mediated immune regulatory network in maize. Further research is needed to comprehensively understand how these elements interact and contribute to maize defense against pathogens, which would provide a more in-depth understanding of the relationship among *ZmH2B*, photosystem-related pathways, and plant immunity.

PSII is embedded within the thylakoids of the chloroplast, where chloroplast-derived reactive oxygen species (cROS) play pivotal roles in both efficient pattern-triggered immunity (PTI) and effector-triggered immunity (ETI) [47, 48]. ETI-related ROS production predominantly stems from PSII. Su et al. determined that in the signaling pathway downstream of ETI prolongs the activation of *MPK3/MPK6* to induce photosynthetic inhibition, leading to the strong accumulation of cROS. Treatment with flavodoxin from cyanobacteria to reduce the production of cROS in *Arabidopsis* chloroplasts led to the disassembly of PSII, thereby attenuating ETI and reducing plant immunity [49]. By contrast, PTI typically requires electron transfer to PSI, a process that is disrupted in *ferrexodin2* plants [50, 51]. Tognetti et al. determined that flavodoxin increased stress tolerance and reduced cROS production. Functionally, flavodoxin could replace ferredoxin in most electron transfer processes [52].

Accumulating evidence suggests that ROS production is specific to different photosystems between ETI and PTI. This specificity arises from the differential regulation of photosystem electron transport chains under distinct immune signaling contexts, where pathogen-derived effectors or PAMPs can perturb chloroplast redox homeostasis. During the conversion of light energy to chemical energy, any environmental or pathogenic stimuli to the components of chloroplasts may affect photosynthesis and facilitate the production of the byproducts cROS [53, 54]. Notably, Chen et al. found that GhHDA15 and GhSRT1 negatively regulate Verticillium wilt resistance in cotton (*Gossypium hirsutum*) by removing lysine 2-hydroxyisobutyrylation (Khib) and succinylation (Ksuc). The photosystem repair protein GhPSB27 is located in the core hubs of both the Khib- and Ksuc-modified protein networks. The acylated GhPSB27 regulated by GhHDA15 and GhSRT1 can increase the D1 protein content, further enhancing plant biomass, seed yield, and disease resistance by increasing photosynthesis and the production of cROS as byproducts [55]. In summary, the complex interactions among the photosystems, ROS production, and plant immunity are influenced by multiple factors. In subsequent experiments, we will examine how ROS affect *ZmH2B* expression and thus plant immunity.

The functions of histone H2Bs in chromatin modification have been widely studied. Among these, H2Bub1 is an important regulatory mechanism for eukaryotic gene transcription that is essential for plant development. *H2Bub1* deletion mutants were more susceptible to *Pseudomonas syringae* pv. tomato DC3000 (*Pst* DC300) than the wild type, and H2Bub1 affects the expression of genes related to the plant SA signaling pathway, which plays a central role in plant immune responses by regulating R gene expression [56]. Symbiotic arbuscular mycorrhizal fungi (AM) also subvert the plant defense response through the H2Bub1 pathway. The effector proteins of the AM *Rhizophagus irregularis* interact with H2Bs and suppress the levels of H2Bub1 via an undetermined mechanism, thereby downregulating defense-related genes and promoting AM colonization [57]. A role of H2Bub1 in viral pathogenesis has also been reported. The Replication initiator protein (Rep) of *Chilli leaf curl virus* (ChiLCV) interacts with *N. benthamiana* HUB1/HUB2 (NbHUB1/2) in the nuclei of infected cells, which promotes the binding of H2Bub1 to the ChiLCV minichromosome. H2Bub1 on the minichromosome enhances the deposition of H3K4me3 on viral genes, thereby upregulating their transcription [58, 59].

In the future, we plan to focus on DEGs within the photosynthesis-related pathway following *ZmH2B* silencing, which represents a promising avenue for unraveling the complex relationship among *ZmH2B*, photosynthesis, and plant immunity. Subsequently, we will investigate the modification-related genes and the changes in their expression following *ZmH2B* silencing, providing information about the roles of histone H2Bs in post-translational modification in maize. Given the well-documented roles of histone H2B modifications, such as H2Bub1, in eukaryotic gene regulation and plant development, understanding their functions in maize could provide insight into the epigenetic regulatory landscape of this important crop.

## Conclusions

ZmH2B contains a conserved Histone_H2A/H2B/H3 domain. When maize was challenged by *B maydis*, *ZmH2B* was upregulated. ZmH2B plays a positive role in regulating maize resistance to *B maydis*. ZmH2B localized to the nucleus in both *N. benthamiana* and maize protoplasts. Additionally, the DEGs following the silencing of *ZmH2B* were predominantly enriched in photosynthesis-related pathways. Our findings could facilitate the development of novel crop varieties with high and stable yield performance under adverse environmental conditions, thereby helping to address the challenges of modern agriculture and ensuring global food security. 

## Materials and methods

### Construction of phylogenetic tree and multiple sequence alignment

Referring to the evolutionary tree presented in the article of Frédéric's group [1], protein sequences of a total of 34 homologous genes from maize, *Arabidopsis thaliana*, and rice were carefully selected for the subsequent construction of subsequent evolutionary tree. The protein sequences of these 34 genes were retrieved from Ensembl Plants (Ensembl Plants) based on their respective gene numbers. These sequences were then imported into MEGA11. Prior to constructing the phylogenetic tree, a multiple-sequence alignment comparison was carried out using the Muscle algorithm. Subsequently, the neighbor-joining method was employed within MEGA11 to build the phylogenetic tree. To enhance the clarity and interpretability of the results, the R packages ggtree [60] and ggmsa [32] were utilized.

### Plant materials, fungal strain and growth conditions

The experimental materials used in this study included the maize B73 inbred line (wild type) and *N. benthamiana*. A mixture of commercial soil and vermiculite was prepared at a ratio of 3:1. This mixture was then filled into pots, in which maize seeds were sown. Throughout the entire growth cycle, the maize plants were cultivated in a greenhouse with a photoperiod of 14 h of light and 10 h of darkness. The greenhouse was maintained day and night temperatures of 24°C and 20°C, respectively, while the relative humidity was kept within the range of 50%−60%. For *N. benthamiana*, seeds were sown in a blend of commercial soil and vermiculite. Once germinated, individual seedling was carefully transplanted into new pots, and the growth conditions were the same as maize. The *B. maydis* strain employed in this study was strain 4–4-3 stored in our lab. The strain was cultured on oatmeal agar plates and incubated at 25°C until the production of spore was achieved.

### *ZmH2B* gene expression analysis

Fourteen-day-old B73 seeds were inoculated with *B. maydis* through live-spray inoculation. The inoculum used was a suspension of *B. maydis* conidia at a concentration of 1 × 10^5^ conidia per ml. Following inoculation, leaves were harvested at 0 h, 12 h, 24 h, 36 h, 48 h, 72 h, 96 h and 120 h. These leaf samples were then processed for RNA extraction and the extracted RNA was reverse transcribed into cDNA. The transcription level of *ZmH2B* in response to *B. maydis* infection was detected using Real-time Quantitative Reverse PCR (RT-qPCR). To determine the relative expression levels of *ZmH2B*, the 2^−ΔΔCt^ method was employed. All experiments were carried out with three biological replicates to ensure the reliability and reproducibility of the results.

### Construction of Agrobacterium-mediated maize VIGS and VOX plants

The VIGS maize plants were generated following the method described by Beernink et al. [61]. To obtain VOX transient overexpression plants, this method was partially adapted through the following steps: first, the full-length CDS (coding DNA sequence) was obtained. Then, we respectively cloned the first 250 bp of the termination codon and the full-length CDS into pCAMBIA1380-FoMV vector to obtain VIGS and VOX vector via homologous recombination in antisense orientation. The freeze–thaw method was employed to introduce these plasmid constructs into *Agrobacterium tumefaciens* GV3101. The transformed Agrobacterium was cultured overnight in LB liquid medium with shaking. Subsequently, the Agrobacterium cells were pelleted by centrifugation and resuspended in infiltration buffer (10 mM MgSO4, 200 μM acetosyringone) to reach an OD_600_ of 1.0. The prepared *Agrobacterium suspension* was injected approximately 2–3 mm above the coleoptilar node of 5-day-old seedlings. After injection, the plants were grown for additional 14 days to observe any emerging symptoms. The silencing efficiency of *ZmH2B* was evaluated using RT-qPCR, with samples collected from the middle part of the fourth or fifth leaf [62]. After an additional week of cultivation, the fourth to sixth leaves displaying viral symptoms were harvested and placed in a 50 ml tube with drierite desiccant at the bottom. The leaves were lyophilized overnight to ensure complete drying and then stored at −20°C. Rub inoculation is a straightforward technique with an infection rate of nearly 90%, which simplifies the generation of gene-silenced plants. Approximately 100 mg of lyophilized tissue was ground in 50 mM potassium phosphate buffer (pH7.0). Maize leaves were dusted with carborundum and then with the leaf sap solution. Rub-inoculation was carried out by gently rubbing the drop of inoculum over the leaf surface using a gloved finger. After inoculation, the leaves were rinsed with tap water to remove excess carborundum. The inoculated plants were then transferred to the greenhouse and monitored for approximately 21 days to observe symptoms. Subsequently, these plants could be used for living inoculation or spray inoculation using *B. maydis*. All primers used for VIGS and VOX plasmid construction and detection of pathogenic fungal biomass are listed in Table S1.

### Pathogenicity analysis

The spore suspension of *B. maydis* was prepared by adjusting its concentration of 1 × 10^5^ conidia per ml using 0.02% Tween-20. The fourth or fifth maize leaves of maize plants were carefully detached and placed in a petri dish (25 × 25 cm) lined with moist filter paper. For inoculation, 10 μl of the spore droplets were precisely dropped onto the maize leaves. The infected maize leaves were cultivated overnight at 25℃ under a high-humidity environment (95% humidity). Subsequently, they were maintained under a 14-h/10-h light/dark photoperiod. Four to five days after inoculation, the disease incidence was investigated. Photographs of the diseased maize leaves were taken to document the symptoms. The area of the disease spots on the leaves was measured using ImageJ software. Additionally, the biomass of the pathogen was quantified by RT-qPCR. All experiments were carried out with three biological replicates to ensure the reliability and reproducibility of the results.

### ROS burst assay

Fourteen days after FoMV-inoculation, the third leaf of the plant was sampled. A 4 mm perforator was used to punch leaf discs, which were then placed in a 9 cm petri dish filled with 20 ml of sterile water. The petri dish was incubated in the dark overnight to allow the leaf discs to equilibrate. Afterwards, the leaf discs were transferred into 1.5 ml tubes. Each tube contained 100 μl of luminol (Bio-Rad Immun-Star horseradish peroxidase substrate), 1 μl of horseradish peroxidase (HRP), and 1 μl of 1 mM flg22, 1 μl of 0.8 mM chitin or ddH_2_O control. Immediately after the leaf discs were added to the reaction mixture, a Glomax 20/20 luminometer (Promega) was used to collect the luminescent signal. The signal was measured every minute for a total duration of 20 min. To ensure the reliability of the results, each sample was subjected to the assay with three biological replicates.

### Plant materials for transcriptome assay

In accordance with the method detailed in 5.4, B73 plants were rub inoculated with FoMV:V, FoMV:*ZmH2B*, FoMV:*GFP*-VOX, FoMV:*ZmH2B*-VOX. Twelve to fourteen days post rub inoculation, the fourth leaf of FoMV:V control plants and *ZmH2B*-silenced plants was harvested for the purpose of determining the silencing efficiency. Subsequently, plants exhibiting higher silencing efficiency were carefully selected and send to a professional company Novogene for transcriptome sequencing. Both the control group (FoMV:V) and the experimental group (FoMV:*ZmH2B*) were configured with three biological replicates, ensuring the reliability and statistical validity of the experimental data.

### Quantification and statistical analysis

Statistical analysis in this study was performed using GraphPad Prism v7.0 and Student’s t-test (*P < 0.05, **P < 0.01, ***P < 0.001, ****P < 0.0001). Data represent the mean ± SD. Details of statistical analysis are provided in the figure legends.

## Supplementary Information

Supplementary Material 1: Supplementary Figure. 1 The DNA sequence of *ZmH2B*. *ZmH2B* (GRMZM2G472696) is a 717 bp gene encoding 238 amino acids.

Supplementary Material 2: Supplementary Figure. 2 The relative expression level of *ZmH2B* was determined after rub inoculation. a. Silencing efficiency of FoMV:*ZmH2B *plants was determined after rub inoculation. b. Transient overexpression efficiency of FoMV:*ZmH2B*-VOX plants was determined after rub inoculation.

Supplementary Material 3: Supplementary Figure. 3 The expression of PR genes in *ZmH2B*-silenced materials FoMV:*ZmH2B *and *ZmH2B*-overexpressed materials FoMV:*ZmH2B*-VOX following infection with *B. maydis*. a, RT-qPCR analyses showing the expression of *ZmPR1*, *ZmPR3*, *ZmPR4*, *ZmPR5* and *ZmPR10* in FoMV:*ZmH2B *following infection with *B. maydis*. b, RT-qPCR analyses showing the expression of *ZmPR1*, *ZmPR3*, *ZmPR4*, *ZmPR5* and *ZmPR10* in FoMV:*ZmH2B*-VOX following infection with *B. maydis*. Data contain mean ± standard error of three replicates (* *p* ≤ 0.05; ** *p* ≤ 0.01; *** *p* ≤ 0.001).

Supplementary Material 4: Supplementary Figure. 4 Analysis of hotmap from *ZmH2B*-silenced plants. a. Silencing efficiency of FoMV:*ZmH2B *plants was determined after rub inoculation. b. The protein heterodimerization activity pathway was analyzed by heatmap. Subsequently, samples with high silencing efficiency were selected for transcriptome sequencing. ZmH2B (GRMZM2G472696) is highlighted in red.

Supplementary Material 5: Table S1 primer used in this study.

## Data Availability

The datasets generated and analysed during the current study are available in the NCBI repository, GSE290889. Availability of data and materials: the Histone H2B protein sequences of maize, rice and *Arabidopsis thaliana* genomes were downloaded from NCBI (National Center for Biotechnology Information). The raw data presented in the article and Supplementary materials are available from the corresponding author on reasonable request.
