# Supplementary material for: Histone ZmH2B regulates resistance to the Southern corn leaf blight pathogen Bipolaris maydis in maize
Source: BMC Plant Biol. 2025 Aug 19;25:1097. doi: 10.1186/s12870-025-07020-9 (PMC12362849; doi:10.1186/s12870-025-07020-9)
Supplement: Supplementary file 1 — Supplementary Material 1: Supplementary Figure. 1 The DNA sequence of ZmH2B. ZmH2B (GRMZM2G472696) is a 717 bp gene encoding 238 amino acids. [file 12870_2025_7020_MOESM1_ESM.pdf]

ATGTCTGGGCCCACATCCACCACGTCAGCGATCCGCATCGCATCCGTCGTCCGGAACGGCCGAACCCCGTCGAACTCC
TCCACGTCAGCGATCCGCGTCGCATATCCACCCACAAATGGCGTCCAACCTCCCGCGCTCTATAAAGTGGCGCCCCCAG
CAGCCTTCTTTCGCACCCAGCAACAAGCAACCTCGTCCCCAAACAAGCAACCACCGCCTCCCCGATCTCGTCGAGAGGA
AGCCCAATCCCGAAACCGCCGCCCTATTCCAATGGCGCCCAAGGCCGAGAAGAAGCCCGCCGCCAAGAAGCCTGCGG
AGGAGGAGCCGGCGGCGGAGAAGGCCACGGCGGGGAAGAAGCCCAAGGCTGAGAAGCGGCTCCCCGCGGGCAAGTC
CGCAGGCAAGGAGGGTGGCGAGAAGAAGGGGAAGAAGAAGGCAAAGAAGTCGGTGGAGACGTACAAGATCTACATCT
TCAAGGTGCTGAAGCAGGTGCACCCGGACATTGGCATCTCGTCCAAGGCCATGTCCATCATGAACTCCTTCATCAACGA
CATCTTCGAGAAGCTGGCGGCGGAGGCGGCCAAGCTGGCGCGGTACAACAAGAAGCCTACCATTACGTCCCGCGAGAT
CCAGACCTCCGTTCGCCTCGTCCTCCCCGGCGAGCTCGCCAAGCACGCCGTCTCCGAGGGCACCAAAGCCGTCACCAA
GTTCACCAGCTCCTAG

Supplementary Figure.1 The DNA sequence of *ZmH2B*. *ZmH2B* (GRMZM2G472696) is a 717 bp gene encoding 238 amino acids.
