# Supplementary material for: Histone ZmH2B regulates resistance to the Southern corn leaf blight pathogen Bipolaris maydis in maize
Source: BMC Plant Biol. 2025 Aug 19;25:1097. doi: 10.1186/s12870-025-07020-9 (PMC12362849; doi:10.1186/s12870-025-07020-9)
Supplement: Supplementary file 2 — Supplementary Material 2: Supplementary Figure. 2 The relative expression level of ZmH2B was determined after rub inoculation. a. Silencing efficiency of FoMV:ZmH2B plants was determined after rub inoculation. b. Transient overexpression efficiency of FoMV:ZmH2B-VOX plants was determined after rub inoculation. [file 12870_2025_7020_MOESM2_ESM.pdf]

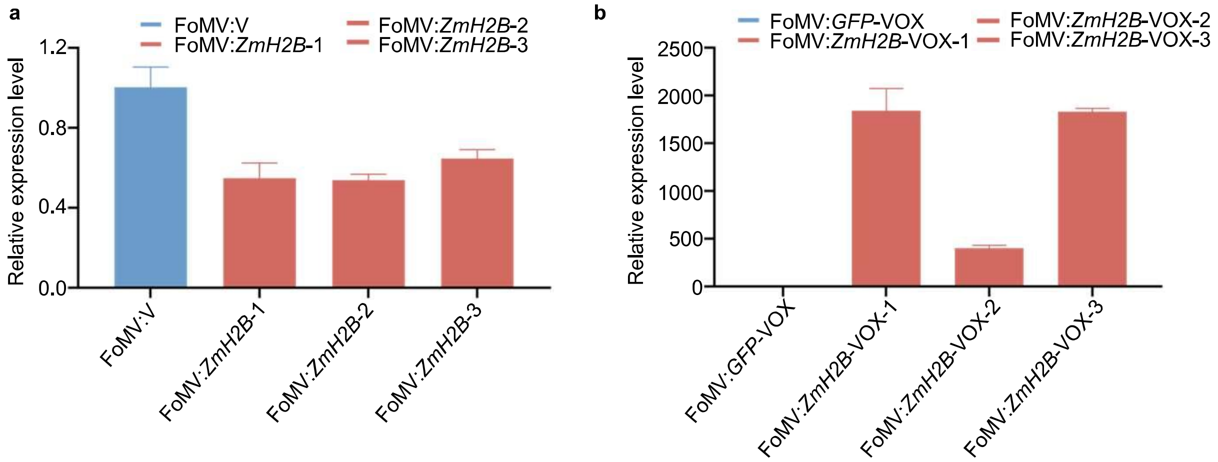

Supplementary Figure.2 The relative expression level of *ZmH2B* was determined after rub inoculation. a. Silencing efficiency of FoMV:*ZmH2B* plants was determined after rub inoculation. b. Transient overexpression efficiency of FoMV:*ZmH2B*-VOX plants was determined after rub inoculation.
