# Supplementary material for: Histone ZmH2B regulates resistance to the Southern corn leaf blight pathogen Bipolaris maydis in maize
Source: BMC Plant Biol. 2025 Aug 19;25:1097. doi: 10.1186/s12870-025-07020-9 (PMC12362849; doi:10.1186/s12870-025-07020-9)
Supplement: Supplementary file 3 — Supplementary Material 3: Supplementary Figure. 3 The expression of PR genes in ZmH2B-silenced materials FoMV:ZmH2B and ZmH2B-overexpressed materials FoMV:ZmH2B-VOX following infection with B. maydis. a, RT-qPCR analyses showing the expression of ZmPR1, ZmPR3, ZmPR4, ZmPR5 and ZmPR10 in FoMV:ZmH2B following infection with B. maydis. b, RT-qPCR analyses showing the expression of ZmPR1, ZmPR3, ZmPR4, ZmPR5 and ZmPR10 in FoMV:ZmH2B-VOX following infection with B. maydis. Data contain mean ± standard error of three replicates (* p ≤ 0.05; ** p ≤ 0.01; *** p ≤ 0.001). [file 12870_2025_7020_MOESM3_ESM.pdf]

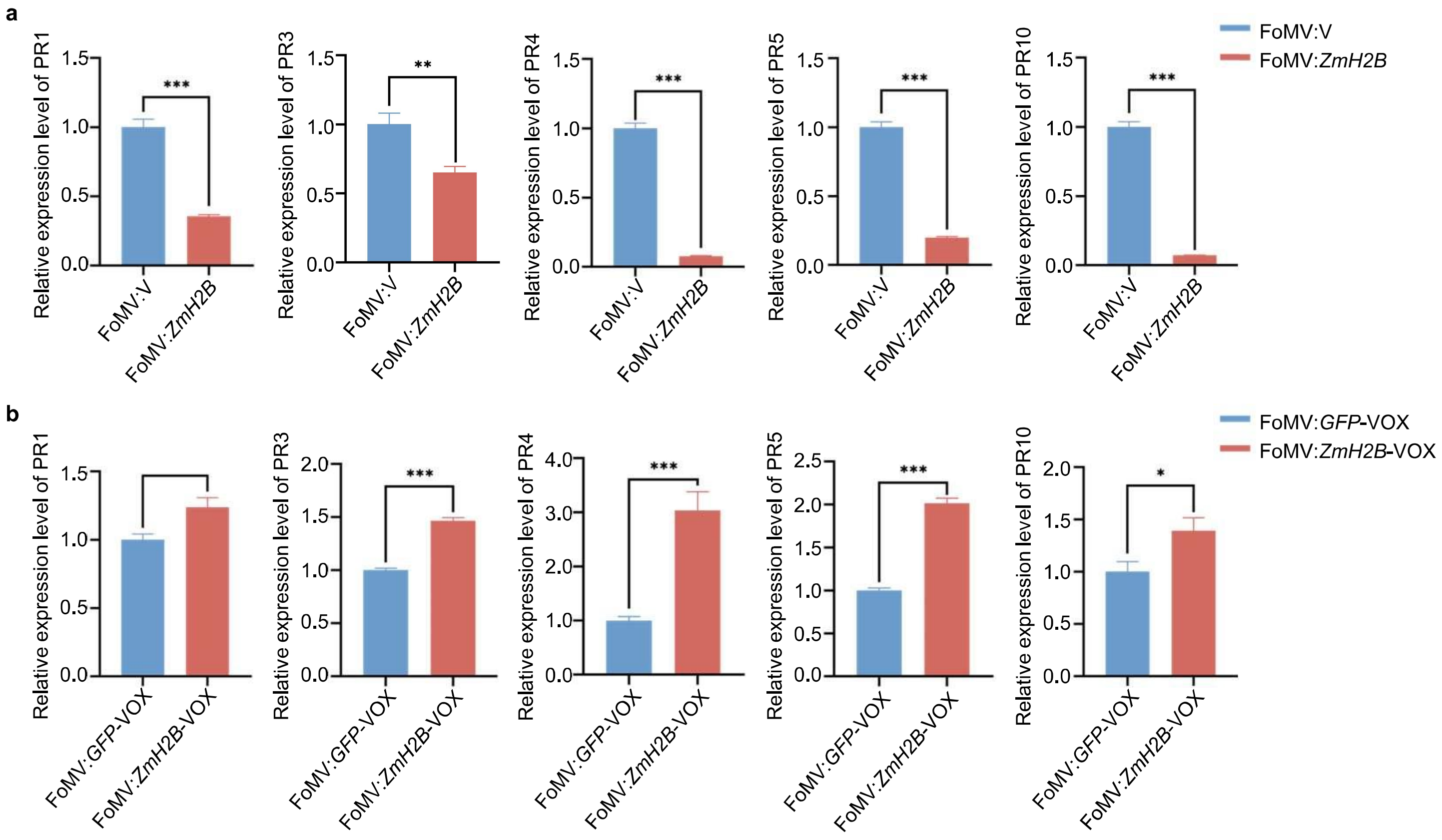

Supplementary Figure.3 The expression of PR genes in *ZmH2B*-silenced materials FoMV:*ZmH2B* and *ZmH2B*-overexpressed materials FoMV:*ZmH2B*-VOX following infection with *B. maydis*. a, RT-qPCR analyses showing the expression of *ZmPR1*, *ZmPR3*, *ZmPR4*, *ZmPR5* and *ZmPR10* in FoMV:*ZmH2B* following infection with *B. maydis*. b, RT-qPCR analyses showing the expression of *ZmPR1*, *ZmPR3*, *ZmPR4*, *ZmPR5* and *ZmPR10* in FoMV:*ZmH2B*-VOX following infection with *B. maydis*. Data contain mean ± standard error of three replicates (* $p \leq 0.05$; ** $p \leq 0.01$; *** $p \leq 0.001$).
