# Supplementary material for: Histone ZmH2B regulates resistance to the Southern corn leaf blight pathogen Bipolaris maydis in maize
Source: BMC Plant Biol. 2025 Aug 19;25:1097. doi: 10.1186/s12870-025-07020-9 (PMC12362849; doi:10.1186/s12870-025-07020-9)
Supplement: Supplementary file 4 — Supplementary Material 4: Supplementary Figure. 4 Analysis of hotmap from ZmH2B-silenced plants. a. Silencing efficiency of FoMV:ZmH2B plants was determined after rub inoculation. b. The protein heterodimerization activity pathway was analyzed by heatmap. Subsequently, samples with high silencing efficiency were selected for transcriptome sequencing. ZmH2B (GRMZM2G472696) is highlighted in red. [file 12870_2025_7020_MOESM4_ESM.pdf]

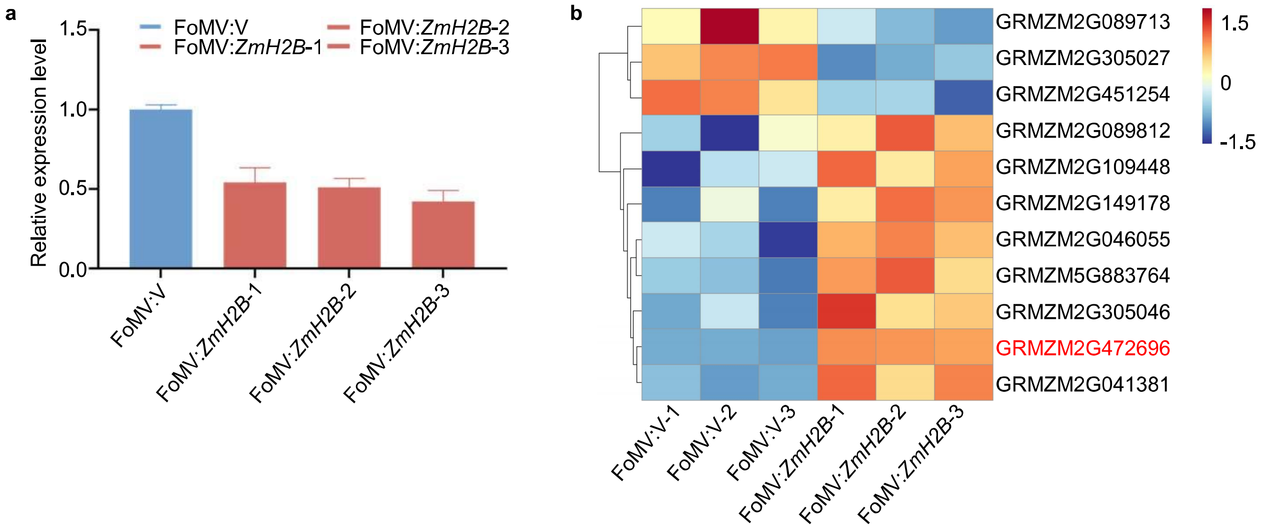

Supplementary Figure.4 Analysis of hotmap from *ZmH2B*-silenced plants. a. Silencing efficiency of FoMV:*ZmH2B* plants was determined after rub inoculation. b. The protein heterodimerization activity pathway was analyzed by heatmap. Subsequently, samples with high silencing efficiency were selected for transcriptome sequencing. ZmH2B (GRMZM2G472696) is highlighted in red.
